# Early neoplastic lesions of the pancreas: initiation, progression, and opportunities for precancer interception

**DOI:** 10.1172/JCI191937

**Published:** 2025-07-15

**Authors:** Brian A. Pedro, Laura D. Wood

**Affiliations:** 1 Department of Pathology, and; 2 Department of Oncology, Johns Hopkins University School of Medicine, Baltimore, Maryland, USA.

## Abstract

Pancreatic ductal adenocarcinoma (PDAC) is known to progress from one of two main precursor lesions: pancreatic intraepithelial neoplasia (PanIN) or intraductal papillary mucinous neoplasm (IPMN). The poor survival rates for patients with PDAC, even those diagnosed with localized disease, highlight the need for pancreatic cancer interception at the precursor stage. Although their basic biological drivers are well characterized, practical strategies for PanIN and IPMN interception remain elusive due to difficulties with detection, risk stratification, and low-morbidity intervention. Recently, advances in liquid biopsy, spatial multiomics analysis, and machine learning technology have provided deeper understanding of the molecular landscapes underlying pancreatic precursor development and progression. In this Review, we outline the different histologic phenotypes, clinical characteristics, and neoplastic cell–intrinsic and –extrinsic drivers of PanINs and IPMNs, with particular focus on current and potential future opportunities for pancreatic precancer interception.

## Introduction

Pancreatic ductal adenocarcinoma (PDAC) is a devastating disease with a dismal overall prognosis (1, 2). The sobering statistics regarding PDAC survival, even for those with early-stage disease, highlight the crucial importance of early PDAC interception. Speaking broadly, cancer interception aims to identify and halt the progression of cancer before the disease can affect patient survival (3). For some cancer types, such as many breast, thyroid, or prostate carcinomas, interception of localized disease through surgical resection and/or targeted therapy is sufficient to mitigate any significant impact on long-term survival (4). By contrast, metastatic capability is believed to arise relatively early during PDAC progression, and PDAC therefore carries significant risk even to patients with localized disease, with five-year survival of only 44% (4, 5). Thus, clinically impactful PDAC interception strategies would ideally be carried out at the precursor stage, before the development of invasive disease.

The vast majority of PDACs progress from one of two major types of precursor lesions — pancreatic intraepithelial neoplasia (PanIN) and intraductal papillary mucinous neoplasm (IPMN) — yet viable approaches for pancreatic precancer interception remain largely elusive. For example, while IPMNs are radiologically detectable, most PDACs are believed to arise from PanINs that are microscopic by definition (6, 7) and are at this time only identifiable within formalin-fixed, paraffin-embedded tissue sections. This microscopic nature of PanINs presents multiple challenges in PDAC diagnosis and research. Firstly, even in high-risk patients undergoing screening via endoscopic ultrasound (EUS) or magnetic resonance imaging (MRI), most PanINs (whether low- or high-grade) are essentially undetectable by imaging. Secondly, researchers’ ability to track and characterize the natural progression of human PanINs is limited, as they can typically only be studied at fixed time points after resection. Additionally, while low-grade pancreatic precancers are highly prevalent (8, 9), the location and anatomic properties of the pancreas mean that intervention strategies carry significantly more morbidity than, for example, colon polyp removal. This underscores the need to identify those lesions that are truly high risk and warrant intervention.

The basic molecular drivers associated with initiation and progression of PanINs and IPMNs have been well characterized through bulk collection and sequencing techniques, providing important insight into their underlying biology. However, there remain critical knowledge gaps that have thus far prevented the development of diagnostic and therapeutic strategies for PDAC interception. Only recently, with the rise of advanced machine learning techniques and high-resolution spatial molecular profiling, have researchers begun to comprehensively study the precise events driving progression of PDAC precursor lesions within the context of their native tissue. In this Review, we outline the known phenotypes, molecular drivers, and mechanisms of progression of the most common PDAC precursors — PanINs and IPMNs — and highlight potential opportunities for pancreatic precancer interception.

## PanIN — histology and molecular characteristics

**Figure 1:**
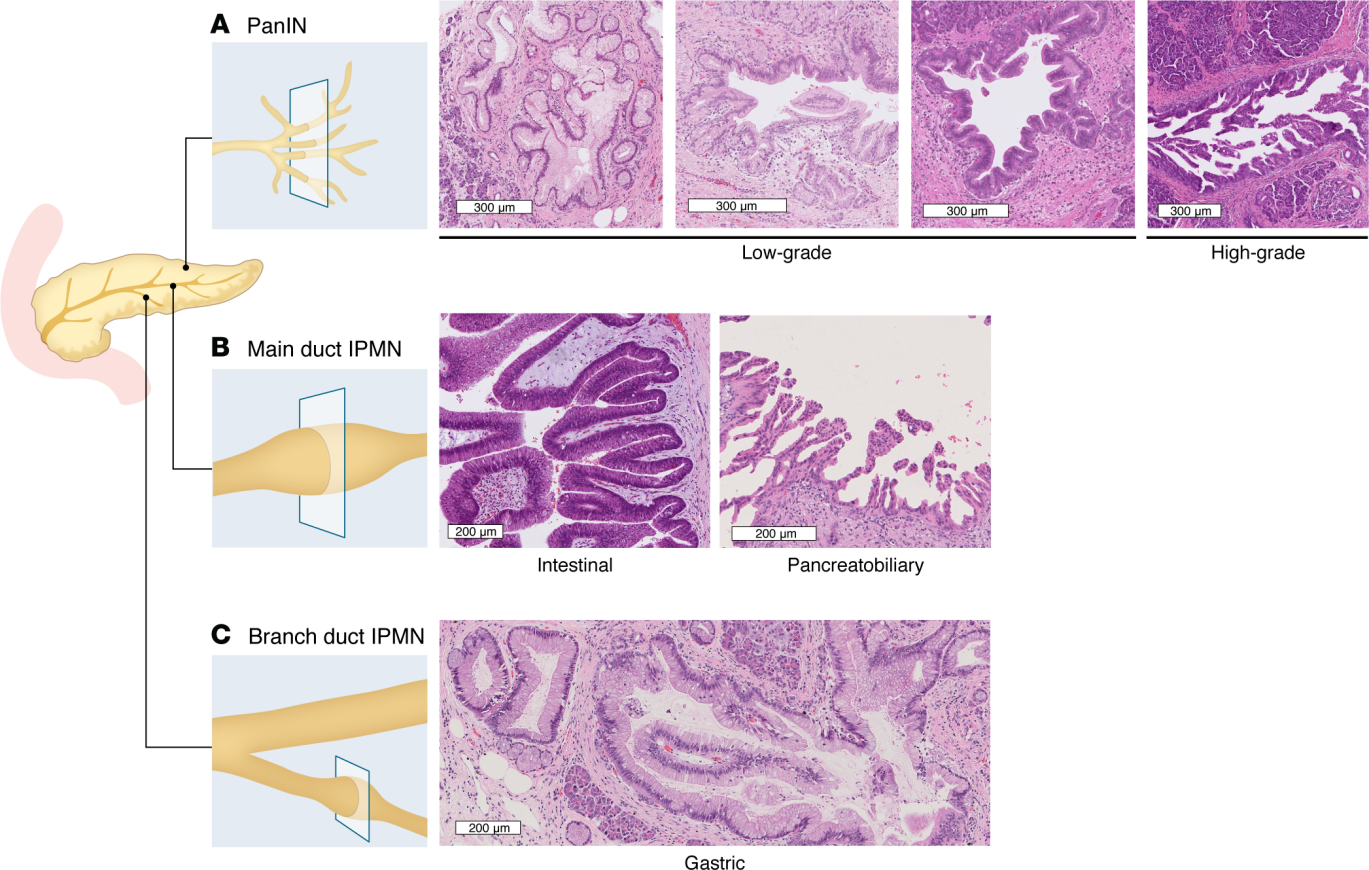
PanIN and IPMN anatomy and histological features. (**A**) PanINs arise from small branches of the pancreatic ductal system and/or foci of acinar-to-ductal metaplasia. Representative H&E cross-sectional images of progression from low-grade to high-grade dysplasia are shown. (**B** and **C**) IPMNs involve the main pancreatic duct (most commonly intestinal or pancreatobiliary types) or side-branch ducts (most commonly gastric type). Intestinal and pancreatobiliary-type IPMNs are more likely to display high-grade dysplasia, while gastric IPMNs more commonly show low-grade dysplasia. Representative H&E cross-sectional images of the three histologic subtypes are shown. Scale bars: 300 μm (**A**) and 200 μm (**B** and **C**).

PanINs, first described by Hulst over 100 years ago (10), are microscopic neoplastic lesions that arise in pancreatic ducts, characterized by mucinous epithelium with varying degrees of dysplasia. PanINs are currently defined as being smaller than 0.5 cm in the longest dimension on a standard histological section; recent 3D reconstructions of human pancreatic tissue challenge the validity of this definition, showing that the complex, branching architecture of PanINs (Figure 1) makes it difficult to accurately determine their size and spatial orientation in standard 2D sections (8, 11). The cell of origin of PanINs is still a topic of debate, although different studies have shown that PanINs can arise from both preexisting ductal cells, or from foci of acinar-to-ductal metaplasia (ADM). Recent evidence predominantly supports the latter, with epigenetic profiling indicating that PanINs possess an intermediate methylation profile that falls between normal ducts and PDAC and is more similar to acinar cells than ductal cells (12–16). Mouse models have further defined two distinct pathways for PDAC initiation: one in which PDAC arises from acinar cell–derived PanINs, and another wherein ductal epithelial cells give rise to PDAC directly without a PanIN intermediate (17–24).

Current classifications, based on the 2014 Baltimore Consensus Meeting for Neoplastic Precursor Lesions in the Pancreas, separate PanINs into low-grade (LG-PanIN) and high-grade (HG-PanIN), with the LG category encompassing lesions historically classified as intermediate-grade (25). Histologic features distinguishing LG-PanIN from non-neoplastic ducts include papillary or micropapillary architecture, columnar epithelium with intracytoplasmic mucin, and cytologic atypia ranging from absent to mild (including loss of nuclear polarity, nuclear enlargement, hyperchromasia, or pseudostratification). By contrast, HG-PanIN displays more complex papillary architecture with possible cribriforming or tufting, along with severe cytologic atypia and mitotic activity (Figure 1) (25). Despite the commonly held theory that PanINs increase linearly with age, recent studies, notably including a cohort of presumed normal donor pancreata, have demonstrated that PanINs are very common even in younger individuals (9). Furthermore, in a cohort of 46 slabs of normal pancreas assessed via 3D modeling, Braxton et al. found that LG-PanINs were highly prevalent across the study population and within individual samples, with a mean of 13 PanINs per cubic centimeter of normal pancreas (8, 9). Importantly, although approximately 85% of PDACs are estimated to arise from PanINs based on histological observations and genetic analyses (26, 27), the vast majority of LG-PanINs never progress to HG-PanIN or PDAC (28, 29). By contrast, HG-PanINs are far rarer (seen in less than 5% of benign pancreata) and are often found in association with invasive PDAC (25, 30–32). Notably, studies involving high-risk individuals (HRIs) have demonstrated a higher overall PanIN prevalence with more frequent HG lesions. Indeed, one study showed a greater than 17-fold increase in mean PanIN lesions in benign pancreata from HRIs compared with age-matched controls; another revealed a nearly 3-fold increase in overall PanINs, and a nearly 5-fold increase in HG-PanINs, in pancreata from patients with familial versus sporadic pancreatic cancer (33, 34).

**Figure 2:**
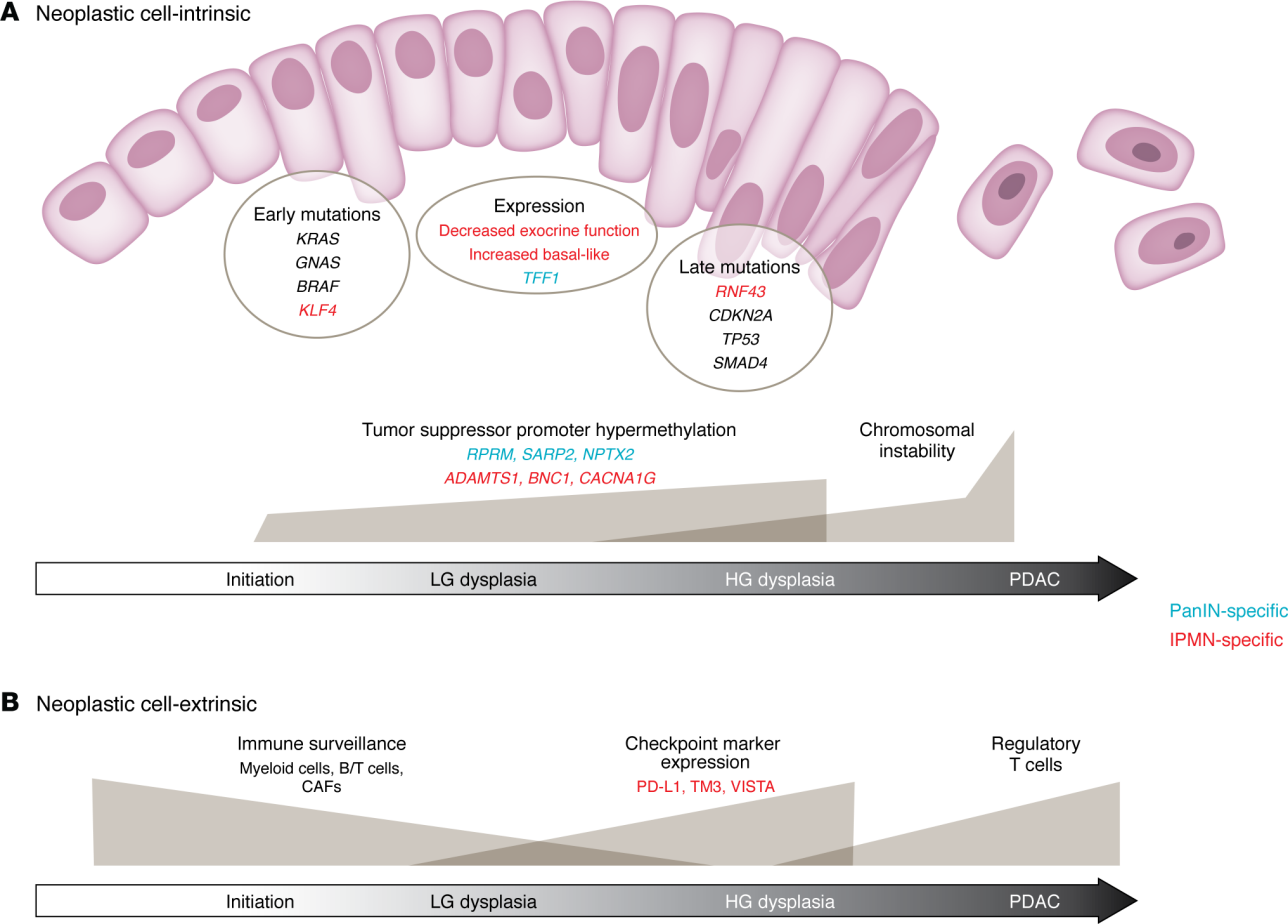
Drivers of pancreatic precancer progression. Schematic of known neoplastic cell–intrinsic and –extrinsic drivers of pancreatic precursor lesion progression from initiation to invasive disease, based on data from studies involving PanINs and IPMNs. (**A**) Early driver mutations are found in oncogenes (most commonly *KRAS*), while later driver mutations occur in tumor suppressor genes. Gene expression drivers of IPMN progression include decreased exocrine function and increased basal-like expression programs, with increased expression of specific genes such as *TFF1* that are associated with PanIN progression. Tumor suppressor promoter hypermethylation is evident even in low-grade lesions, with gradual increases with increasing grade. Chromosomal instability is evident in high-grade lesions but shows an abrupt increase in invasive disease. (**B**) Immune surveillance decreases and the microenvironment becomes more immunosuppressive with increasing grade, including increased expression of checkpoint markers and higher density of regulatory T cells.

The basic biology of PanINs has been extensively characterized, with *KRAS* hotspot mutations recognized as a virtually universal early event (Figure 2) (35). Identification of specific *KRAS* variants can therefore aid in differentiating PanINs within the same specimen, although there is some evidence that a single PanIN can arise polyclonally (8). In the rarer cases of *KRAS* wild-type PanINs, alternative early driver mutations include *GNAS* or *BRAF* (35). Progression to HG-PanIN is associated with *CDKN2A* and/or *TP53* inactivation, while inactivation of *SMAD4* is typically seen in the context of associated PDAC and is only rarely found in isolated HG-PanIN (36). While some studies have shown *SMAD4* loss in HG-PanINs, this is considered a late event in PanIN progression. These cases also most commonly contain invasive PDAC, raising the possibility of cancerization of ducts mimicking HG-PanIN (37, 38). Due to the microscopic nature of PanINs, molecular characterization has historically been through bulk collection (e.g., laser capture microdissection) and subsequent genomic and transcriptomic analyses. Bulk characterization is effective in defining overall genetic profiles of PanIN lesions and performing larger-scale comparisons between multiple PanINs and/or invasive PDAC within single specimens. However, this approach largely precludes a more nuanced investigation of the spatially and temporally defined genetic events that drive progression of a fraction of LG-PanINs.

Recently, advances in spatial transcriptomics and other similar techniques have allowed for more precise correlation between molecular changes and specific cellular phenotypes, as well as consideration of such changes in the context of the tissue microenvironment. Notably, Carpenter et al. found that within a cohort of donor pancreata, sporadic PanINs show predominantly similar gene expression profiles compared to established signatures from PDAC and PDAC-associated PanIN, an indication that even LG-PanIN lesions may have already acquired many features of malignancy (9). Bell et al. demonstrated a defined, gradual increase in expression of *TFF1*, a gene known to be overexpressed in both PanIN and PDAC, as LG-PanIN progresses to HG-PanIN (16). Furthermore, they showed that progression of HG-PanIN to PDAC is accompanied by increased proliferative capacity and decreased cancer-associated fibroblast–related (CAF-related) inflammatory signaling (16). At the DNA level, multiple studies have demonstrated a relatively abrupt, late accumulation of chromosomal changes as LG-PanIN progresses to HG-PanIN and PDAC, often including at least one chromothripsis event (8, 36, 39–41). As discussed above, methylation profiling of PanINs indicates an intermediate epigenetic state between normal ducts and PDAC, with more similarities to acinar cells than ductal cells. This suggests that epigenetic priming may help to facilitate progression from PanIN to PDAC, and it further supports the hypothesis that PanIN arises in the context of ADM (14, 16). Specifically, increased promoter hypermethylation in tumor suppressor genes, including *RPRM*, *SARP2*, and *NPTX2*, among others, has been correlated with increasing PanIN grade (42).

Aside from cell-intrinsic factors, the surrounding tissue microenvironment and immune cell milieu are perhaps equally as important to the initiation and progression of pancreatic precursor lesions. The ability of neoplastic cells to evade the host immune response has been recognized as a hallmark of cancer for over a decade (43, 44), yet challenges remain in characterizing the immune cell environment for different precancers and cancer types. This is particularly true for PanINs, given their small size and complex architecture. However, recent advances in machine learning, 3D tissue modeling, and spatial multiomics techniques have allowed for more holistic quantification of the immune cell response to PDAC precursors at various stages along their progression. Bell et al. applied a novel pipeline to a cohort of matched LG- and HG-PanINs and found that even LG lesions are accompanied by CAF subtypes typically seen in the PDAC microenvironment, including myofibroblastic CAFs, inflammatory CAFs, and antigen-presenting CAFs (16). This suggests that protumorigenic remodeling of the tumor microenvironment begins even in early precursor lesions (16). They further showed that the CAF-related inflammatory signaling in PanINs decreases during PDAC invasion and is accompanied by increased proliferation-related signaling, consistent with the densely fibrotic and immunosuppressive microenvironment that is characteristic of PDAC (45–49). Carpenter et al. used multiplex immunofluorescence to quantify the PanIN microenvironment in otherwise normal pancreata, finding that the stroma associated with PanINs preferentially contained myeloid cells, CD4^+^ T cells, activated fibroblasts, and collagen, compared with normal acinar tissue (9). Importantly, PanINs lacked regulatory T cells (Tregs), whereas invasive PDAC was found to be rich in Tregs, suggesting that immune surveillance in precursor lesions gives way to a more immunosuppressive microenvironment at the later stages of neoplastic progression (9).

## IPMN — histology and molecular characteristics

IPMNs, as the most common pancreatic cyst and second most common PDAC precursor lesion (believed to give rise to <10% of PDACs overall) (7, 50), present distinct molecular characteristics and clinical implications. While substantial histologic overlap exists between PanINs and IPMNs, IPMNs overall are larger (>1 cm), typically more architecturally complex, and subclassified by location (main duct, branch duct, or mixed) and histologic subtype (gastric, intestinal, or pancreatobiliary) in addition to grade of dysplasia (HG or LG, analogous to PanIN; Figure 1) (25). Although the vast majority of IPMNs do not progress to PDAC, with overall risk of malignant transformation estimated at 33 per 100,000 cases, main-duct IPMNs carry up to 65% risk of malignant transformation (51). Gastric-type IPMNs are most commonly LG and derived from branch ducts, while intestinal and pancreatobiliary IPMNs more often involve the main duct and display HG dysplasia (52).

The macroscopic size of IPMNs allows for radiographic detection and risk stratification prior to potential surgical resection. While larger cysts can produce clinical symptoms leading to diagnosis, IPMNs are most often discovered incidentally on CT or MRI scans (52, 53). Indications for IPMN resection, rather than active surveillance, include main duct dilation, presence of mural nodule(s) or an associated mass, malignant cytology, or patient symptoms such as jaundice or clinical pancreatitis (52, 54). Larger cysts (>3 cm) without additional high-risk features are often approached with shorter-interval follow-up radiologic screening, transitioning to standard-frequency screening if no significant increase in size is observed (52, 54). Additional clinical history, such as family history of IPMN or PDAC, or known germline mutations associated with higher PDAC risk, are also considered when deciding whether and how frequently to screen for IPMNs; notably, a retrospective study by Skaro et al. demonstrated that patients with certain PDAC-associated germline mutations have increased risk of concurrent invasive carcinoma within IPMN resection specimens (55). Genetic predisposition is believed to contribute to IPMN development, given the frequent multifocality of IPMNs, tendency for additional IPMNs to arise in the remnant pancreas following resection, and increased risk for extrapancreatic neoplasia among IPMN patients (56–60).

Like PanINs, the majority of IPMNs (60%–80%) harbor *KRAS* hotspot driver mutations. However, other early IPMN driver mutations in genes such as *GNAS* (50%–70% of IPMNs) and *KLF4* (21%–53% of IPMNs) are much rarer in PanINs (Figure 2) (30, 61, 62). Additional changes in *RNF43*, *CDKN2A*, and *TP53* are believed to arise later in IPMN progression to HG dysplasia or invasive disease. Notably, *GNAS* and *RNF43* changes are typical of PDAC arising from IPMN, but not of PDAC that lacks an associated IPMN (30). The specificity of *GNAS* mutations for IPMNs is especially useful in cyst fluid analysis, where they are considered diagnostic for IPMN (30). Interestingly, genomic analysis of entire IPMNs has shown that driver mutations in certain genes, including *KLF4* and *RNF43*, can lead to clonal expansion within only LG lesions, while these clones are then selected against during IPMN progression (61, 63). Instead, HG lesions display multiple mutations in later driver genes, thereby suggesting a model of IPMN progression that combines early clonal selection with later convergent evolution (61, 63, 64). As with PanINs, epigenetic changes are also thought to contribute to IPMN progression. One study demonstrated increased promoter hypermethylation of the tumor suppressor genes *ADAMTS1*, *BNC1*, and *CACNA1G* in IPMN-advanced neoplasia versus LG-IPMNs (65).

The IPMN microenvironment displays stark differences between LG and HG or invasive lesions. Hernandez et al. applied multiplex immunofluorescence to human IPMN tissue to demonstrate that immune cell infiltration decreases as lesions progress from LG to HG to PDAC (66). Indicators of ongoing immune surveillance, including activated B cells and cytotoxic and memory T cells, were found at higher density in LG-IPMNs, while decreased immune surveillance was evident in certain LG-IPMNs that eventually progressed to HG-IPMN. This contrast highlights the potential role of immune infiltration in preventing IPMN progression (66). These findings are consistent with those from Roth et al., who performed comprehensive IHC assays across a range of human IPMN resection specimens to show that the active, T cell–rich microenvironment of LG-IPMNs becomes immunosuppressive and Treg dominant in invasive PDAC (67). Similarly, Jamouss et al. recently showed via IHC that progression to HG-IPMNs is accompanied by fewer cytotoxic T cells, increased macrophage density (specifically in HG foci), and overall increased expression of immune checkpoint markers, including PD-L1, TIM3, and VISTA (68). Overall, these findings suggest a potential role for immunomodulatory approaches to IPMN interception, taking advantage of the proimmunogenic microenvironment that is characteristic of LG lesions.

## Approaches to PDAC interception at the precursor stage

Given the relative rarity of PDAC among the general population (13.5 estimated new cases per 100,000 individuals in the United States per year) (4), routine screening is not recommended (69). Instead, detection of PDAC or precursor lesions is either triggered by patient symptoms or occurs incidentally via unrelated radiographic studies. However, certain HRIs, defined either by family history (≥2 first-degree relatives with PDAC) or germline mutations in certain DNA repair–related genes, including *BRCA1/2*, *PALB2*, and *ATM*, are known to develop PDAC at higher rates than the general population (70). Depending on the clinical scenario, the American Gastroenterological Association recommends surveillance screening via MRI and/or EUS beginning at either 40 years, 50 years, or 10 years younger than the initial familial age of onset (69). These recommendations cite studies showing a significant survival benefit of PDAC screening in HRIs, although there is also a significant risk of morbidity from subsequent surgical procedures, and current radiological techniques are largely unable to detect PanIN lesions. While few pancreatic precancer interception strategies are currently employed, new advances in high-risk biomarker identification have already begun to inform numerous promising avenues.

### PanIN interception.

**Figure 3:**
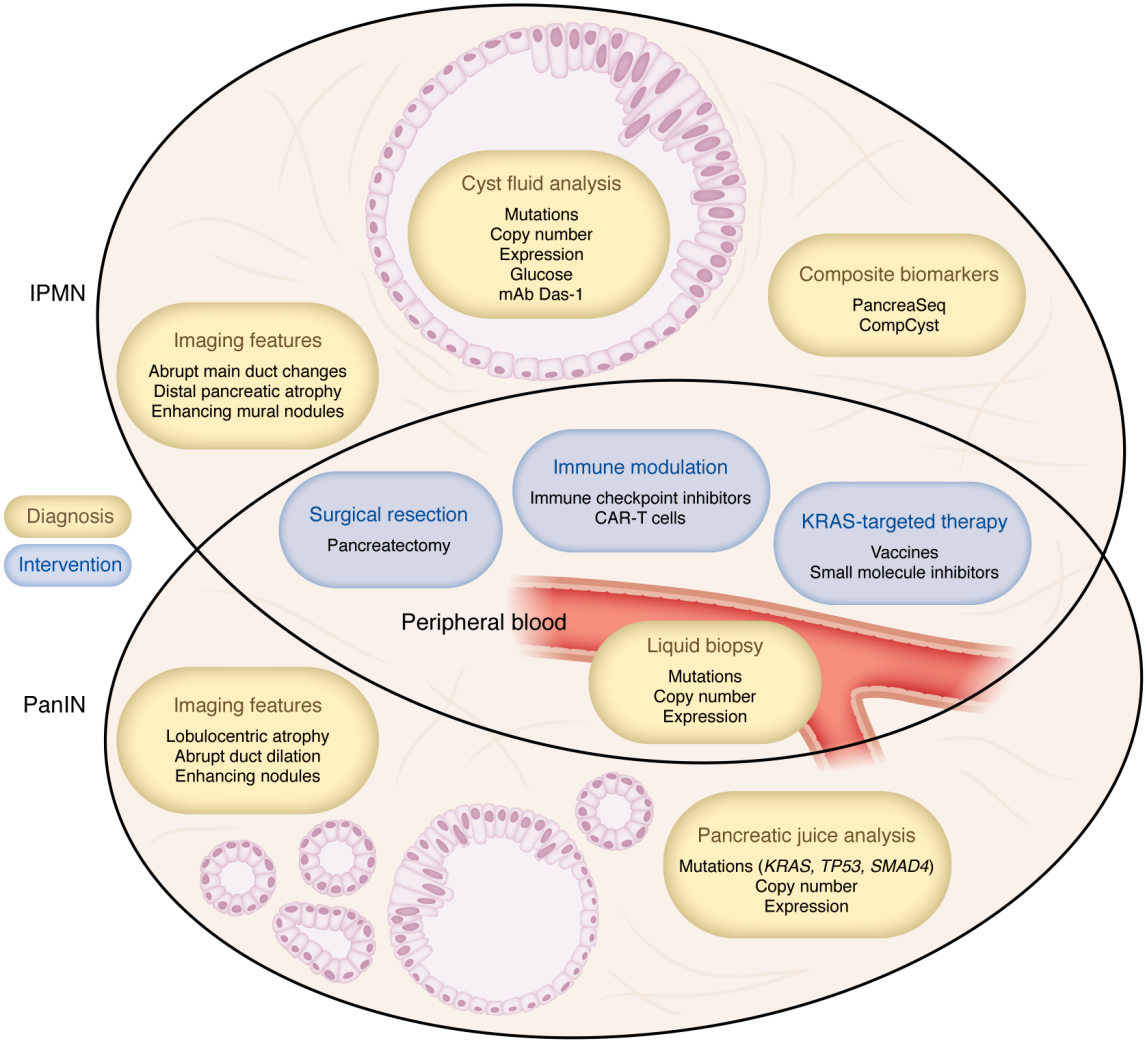
Current and potential opportunities for pancreatic precancer interception. Schematic of current and theoretical strategies for PanIN and IPMN interception, categorized by lesion type and role in interception (diagnosis or intervention). PanIN detection strategies are limited but include certain imaging features and pancreatic juice analysis. Current IPMN detection strategies include imaging and cyst fluid analysis, with multiple composite biomarkers for risk stratification recently becoming available. Liquid biopsy analysis of peripheral blood has not been thoroughly studied for precursor lesions but is theoretically applicable. Potential intervention strategies for both PanINs and IPMNs include surgical resection, immune-based therapies, or *KRAS*-targeted therapies.

The current body of knowledge regarding PanIN progression suggests multiple strategic approaches for PanIN interception (Figure 3). Importantly, modeling studies using sequencing data from PanINs and associated PDAC estimate a substantial time interval, ranging from approximately 4 to 12 years, for PDAC development from either the common ancestral cell or from a HG-PanIN precursor (29, 71), providing a crucial window of time for PDAC interception. Given the heavy predominance of *KRAS* mutations in LG-PanINs, one theoretical approach involves using anti-KRAS therapy to target PanINs before they can progress to HG lesions or PDAC. Indeed, clinical trials are currently ongoing to evaluate the efficacy of KRAS-targeted vaccine-based therapy in pancreatic and other cancers (72, 73). A recent phase I clinical trial study also demonstrated the safety and immunogenicity of a mutant KRAS–targeted vaccine in HRIs prior to PDAC development, highlighting its promise as a novel strategy for precancer interception (74). Furthermore, considerable progress has been made in the development of targeted KRAS inhibitors in recent years, with two KRAS inhibitors already approved by the Food and Drug Administration for use in non–small cell lung cancer, and multiple others in clinical trials (75, 76). However, these therapies have been primarily investigated in the setting of invasive disease, rather than precancer. Ultimately, the practical utility of such a wide-net approach will be dictated by the presence and severity of therapy-related side effects, given the relatively high prevalence of LG-PanINs and rarity of progression to HG-PanIN. For interventions with higher cost and/or potential side-effects, a more logical inflection point to target is the transition from LG- to HG-PanIN, given the presumed large proportion of HG-PanINs that progress to invasive PDAC (29, 30). However, this approach inherently requires a clinically feasible detection strategy, including a measurable, specific diagnostic biomarker of HG-PanIN that has not yet progressed to PDAC.

Although radiographic screening is useful for IPMNs, as discussed in detail later, radiology has not yet proven to have a consequential role in PanIN diagnosis. However, multiple studies have demonstrated a reproducible histologic pattern of lobulocentric atrophy associated with PanINs, particularly in HRIs for whom PanIN burden is often higher, that is detectable by EUS (6, 34). Furthermore, Kiemen et al. leveraged CODA, a novel histology- and machine learning–based pipeline for 3D tissue reconstruction, and subsequent re-review of prior CT scans, to demonstrate that larger PanIN lesions are in fact radiographically visible (11). It is worth noting, however, that many of these lesions may technically exceed the 1.0 cm definition for PanINs when considering their longest 3D axis (11). In recent years, multiple studies have attempted to define radiographic criteria for identification of HG-PanIN. CT and/or MRI features found to be associated with HG-PanIN include abrupt main pancreatic duct changes with distal pancreatic atrophy, enhancing mural nodules 5 mm or larger, and retention cysts/microcysts, although data are conflicting about the ability of the latter to distinguish between LG- and HG-PanIN (77–79). However, these studies are specifically performed in patients with concomitant IPMNs or other pancreatic tumors necessitating imaging studies, potentially limiting the generalizability of their findings for isolated HG-PanIN screening. Given the lack of symptoms associated with PanIN lesions, the patient population most likely to benefit from a possible radiographic screening approach are HRIs for whom routine screening is already recommended (69).

Noninvasive or minimally invasive methods for PDAC screening are also becoming more widely studied and utilized, and these could provide an alternative approach for detection and interception of HG-PanIN. Isolation and analysis of tumor cells or cell-free tumor DNA, RNA, proteins, or extracellular vesicles in circulation or other bodily fluids, also known as liquid biopsy, is a rapidly expanding field for the diagnosis and surveillance of a variety of tumor types, including PDAC (80, 81). These approaches carry multiple advantages, including minimizing potential surgical or procedural complications (80). However, many liquid biopsy techniques are still in the process of being validated and standardized for use in clinical settings, and downstream analysis and diagnosis is limited by the availability of known biomarkers for the disease of interest and sensitivity of testing methods. In addition, cell-free DNA (cfDNA) detection in peripheral blood is likely only applicable in cases of invasive disease where tumor cells have reached the bloodstream, and even then, the levels of detectable cfDNA in early invasive PDAC are typically low (81, 82). Detection of non-invasive precursor lesions would instead require more localized methods, such as pancreatic juice or cyst fluid analysis. While cyst fluid analysis is feasible for IPMNs, the small size of PanINs likely prohibits such an approach. Studies have shown that *KRAS* and *TP53* mutations are detectable in pancreatic juice obtained from the duodenum in patients with PDAC during EUS surveillance (83–88). Importantly, even patients with HG dysplasia as their highest-grade lesion have been found to harbor higher concentrations of mutant *TP53* and *SMAD4* in pancreatic juice, highlighting the potential utility of this approach in detecting HG precursor lesions (89). However, at present, the lack of defined, sensitive biomarkers, and paucity of clinical validation, limits the utility of liquid biopsy as a screening method for HG-PanIN (90, 91). Moving forward, integration of novel tissue modeling techniques and spatial molecular analysis will ideally allow for characterization of precise, detectable biomarkers for preinvasive HG-PanIN lesions.

### IPMN interception.

The larger size of IPMNs compared with PanINs allows for easier detection, and thus a more diverse array of potential interception strategies (Figure 3). However, although an estimated 30% of resected IPMNs eventually demonstrate an invasive component (92–95), the molecular landscape at the transition point from IPMN to invasive PDAC has yet to be comprehensively mapped. Like PanINs, modeling studies based on IPMN sequencing data suggest an approximately 4-year interval between the development of HG dysplasia and invasive PDAC (96). IPMN histologic subtypes each carry some prognostic significance; for example, since gastric-type IPMNs are typically LG, identification of biomarkers for gastric differentiation could help categorize certain IPMNs as lower-risk, avoiding unnecessary intervention. However, the molecular drivers underlying IPMN phenotypes show substantial overlap, complicating the development of specific predictive biomarkers. One study found that *KRAS*, *GNAS*, and *RNF43* mutations were all present within gastric, intestinal, and pancreatobiliary subtypes, although *GNAS* and *RNF43* mutations were most common in intestinal and rarer in pancreatobiliary lesions (97). This is also illustrated by studies involving the *NKX6-2* gene. Sans et al. leveraged spatial transcriptomics analysis of human and mouse IPMN tissue to identify *NKX6-2* as a putative biomarker of lower-risk, gastric-type IPMNs that could feasibly be detected in cyst fluid analysis (98). By contrast, a separate spatial transcriptomics study concluded that *NKX6-2* is a specific marker for HG gastric IPMNs, and thus is actually an indicator of higher-risk lesions (99). Finally, while gastric-type IPMNs are typically considered low-risk, Liffers et al. used methylation and transcriptomic analyses, as well as low-coverage whole-genome sequencing, to show that gastric-type IPMNs express higher *MUCL3* (mucin-like 3) and harbor more recurrent copy number variations than PanINs, both of which could be associated with higher risk of progression (100). The same study also evaluated copy number changes within the different histologic IPMN subtypes, finding higher chromosomal instability among intestinal IPMNs, even in LG lesions (100). Numerous studies have demonstrated the feasibility of copy number variation analysis using cfDNA from liquid biopsy samples in multiple cancer types, including PDAC (81, 101–104), but its presence in LG lesions suggests limited utility as an isolated biomarker for higher-risk lesions. It is also important to consider the possibility of intralesional heterogeneity, with single IPMNs containing a range of grades and histologic subtypes; therefore, any single marker may not be representative of the entire lesion, especially for multiloculated cysts. Some genetic studies have even hypothesized that gastric-type IPMNs are a precursor to intestinal or pancreatobiliary types, while others have postulated a common lineage between gastric and pancreatobiliary types, with intestinal-type arising through a separate pathway (97, 100, 105–107). Overall, these studies highlight the need for biomarkers that more precisely correlate with histologic grade, rather than subtype alone. To this end, a recent study by Iyer et al. analyzed spatial transcriptomic profiles of IPMNs with a range of histologic grades, identifying specific RNA expression profiles that could categorize IPMNs as normal, low-risk, or high-risk, with the high-risk lesions displaying diminished exocrine function and increased basal-like gene expression programs (108).

Importantly, screening for IPMNs is more clinically feasible than for PanINs, and the larger size of IPMNs allows for molecular analyses via cyst fluid collection in many cases. Indeed, measurement of cyst fluid carcinoembryonic antigen (CEA) levels has been extensively investigated as a diagnostic tool to differentiate precancerous mucinous cysts — IPMNs and mucinous cystic neoplasm (MCN, another less common PDAC precursor) — from benign cysts such as serous cystadenomas. Recent data show that combining CEA measurement with next-generation sequencing for common IPMN driver mutations can increase diagnostic accuracy (52, 109). However, CEA is not reliably predictive of the grade of dysplasia within IPMNs (52). A recent meta-analysis examined the predictive ability of certain DNA mutations to identify pancreatic mucinous cysts, finding that *KRAS* and/or *GNAS* mutations were more sensitive and specific than CEA levels (110). Similarly to CEA, however, the authors found that *KRAS* and *GNAS* could not reliably distinguish cysts with or without HG dysplasia or PDAC; other mutations, including *CDKN2A*, *PIK3CA*, *SMAD4*, and *TP53*, were highly specific, but not sensitive for HG dysplasia or PDAC (110). Also currently under investigation is cyst fluid glucose measurement, which shows equivalent or better diagnostic accuracy than CEA with lower associated cost (52, 110). A newer, promising cyst fluid biomarker for IPMN risk stratification is Das-1, a murine mAb developed to react specifically with normal colonic epithelial cells (111, 112). Das et al. have demonstrated that cyst fluid ELISA using mAb Das-1 can identify high-risk IPMNs (i.e., any subtype with HG dysplasia, intestinal-type IPMNs with intermediate-grade dysplasia, or IPMNs with associated invasive carcinoma) with high sensitivity and specificity (110–112). Additionally, preliminary studies measuring MUC5AC protein levels in pancreatic cyst fluid from surgical specimens have shown high sensitivity and specificity for identifying mucinous cysts with HG dysplasia or PDAC, although this marker remains to be tested in the setting of EUS-guided fine needle–aspirated cyst fluid (110, 113–115).

While cyst fluid analyses have shown promise in the diagnosis and classification pancreatic cystic lesions, the inherent molecular complexity of such lesions limits the prognostic utility of any single biomarker. To address this, recent studies have sought to design more precisely predictive panels through integration of multiple molecular and clinical markers. Springer et al. initially demonstrated the ability of molecular features (mutations, loss of heterozygosity, and aneuploidy) to identify different pancreatic cystic lesions, finding that composite molecular and clinical markers performed better than single or composite molecular features alone (116). They further expanded upon this approach with CompCyst, a machine learning–based method to guide pancreatic cyst management that showed greater concordance with gold standard pathologic diagnosis than conventional clinical management approaches (116, 117). Similarly, Singhi et al. and later Nikiforova et al. developed and refined the PancreaSeq targeted DNA/RNA sequencing panel for pancreatic cyst fluid analysis, achieving high sensitivity and specificity for detecting advanced neoplasia through a risk score that integrates characterization of mutations (*GNAS*, *TP53*, *SMAD4*, *CTNNB1*, mTOR pathway genes), mutant allele frequency, copy number changes, gene fusions, and gene expression profiles (118, 119). Moving forward, improvements in molecular characterization techniques and machine learning approaches will allow for development of such composite biomarkers with even greater degrees of complexity and precision.

### Microenvironmental- and immune-based approaches to precursor interception.

As discussed above, the microenvironments of both LG-PanINs and LG-IPMNs display evidence of immune surveillance that transitions to immunosuppression as these lesions progress to HG dysplasia and especially invasive disease (9, 16, 66–68). Importantly, these findings suggest that if timed correctly, proimmunogenic therapies could be deployed to intercept PDAC precursor lesions prior to development of HG dysplasia and invasive cancer. One such approach, using mutant KRAS–targeted vaccines in patients with evidence of cystic pancreatic neoplasia, or those at high risk of PDAC development, is currently being investigated through early-phase clinical trials (73). Although PDAC is known to possess a relatively low tumor mutational burden that seems to limit its responsiveness to immune checkpoint inhibitor (ICI) therapy (120), recent evidence showing increased immune checkpoint marker expression in HG-IPMNs (68) suggests a possible role for ICIs in targeting precursor lesions. Additional immune-based approaches currently under investigation for PDAC treatment, including oncolytic virus therapy and chimeric antigen receptor T cell (CAR-T) therapy, could theoretically be applied to target precursor lesions, especially given the similar molecular profiles between HG precursors and invasive PDAC (45). Overall, application of any immunomodulatory approaches would require careful consideration of the threshold of acceptable off-target effects, which may be lower in the setting of cancer prevention versus active cancer treatment. As with other precursor interception strategies, more precise stratification of precursor lesions through novel biomarkers could aid in identifying higher-risk LG lesions that justify immune-based interventions.

## Discussion and conclusions

Even as advanced analytical methods have vastly broadened our understanding of the molecular and genetic underpinnings of pancreatic precursor lesions, there remains a need for effective, innovative approaches for pancreatic precancer interception. For PanINs, the advent of 3D tissue modeling and spatial multiomics have been revolutionary in more precisely defining the cell-intrinsic and -extrinsic factors leading to their initiation and progression from LG to HG to PDAC. However, PanIN interception strategies are still severely limited by their microscopic nature and lack of measurable, predictive biomarkers for HG lesions. By contrast, IPMNs are more easily detectable by radiology due to their larger size, also making them more amenable to diagnostic approaches such as cyst fluid analysis. Characterization of IPMNs has further been aided by spatial profiling, allowing for more robust comparisons between histologic subtypes that are known to carry different prognoses. However, the complex evolutionary dynamics of IPMN and PanIN progression have largely inhibited the identification of biomarkers for the patients most likely to benefit from more aggressive intervention. Fortunately, novel machine learning approaches are increasingly being utilized to develop high-level, composite biomarkers that can achieve greater levels of prognostic specificity than single mutational biomarkers or simple gene expression panels.

For HRIs, who experience higher rates of PDAC (and precursor lesions) due to known germline mutations or clinically significant family history, standardized routine screening allows for earlier detection of precursors than in the general population. This makes HRIs an ideal group in which to develop new interception strategies, and researchers with access to HRI patient cohorts are thus uniquely positioned to study the progression of PanINs and IPMNs. Furthermore, while the biology underlying the higher incidence of PDAC and its precursor lesions within HRI populations is still being explored, the inherent DNA repair deficiencies resulting from germline *BRCA1/2*, *PALB2*, and *ATM* mutations are reflective of the increased genomic instability observed in HG precursor lesions and PDAC (39–41, 96). As discussed by Chari and others, general population screening for high-risk precursor lesions or PDAC is not cost effective due to the relative rarity of pancreatic cancer (121, 122). Instead, the data that emerge from studying existing HRI cohorts can then be employed to refine the definition of “high-risk,” creating an enriched population of patients whose lesions are more likely to progress to invasive disease based on clinical, molecular, and radiographic features, in addition to family or genetic history. This refined high-risk population could then be screened using novel biomarkers for HG precursor lesions that warrant surgical resection or other intervention approaches. However, potential clinical trials evaluating pancreatic precancer interception still face numerous challenges, including prolonged follow-up duration (given the lengthy estimated time to progression from some precursors), complexity of defining potential endpoints, and lack of precursor lesion–specific criteria for evaluating therapeutic response (analogous to the RECIST system in solid tumors) (123).

One of the defining characteristics of pancreatic precursor lesions is their complex, 3D branching architecture. Although pathologists are still tasked with diagnosing and characterizing these lesions through 2D cross sections, studies have shown that this approach may vastly underestimate the size, and inaccurately estimate the number, of such lesions (8, 11). For example, branches of a single PanIN may appear as spatially distinct PanINs in a single 2D section; conversely, smaller PanINs could be missed if deeper sections of a tissue block are not examined. Moving forward, the integration of spatial multiomics approaches with newly developed 3D modeling techniques will provide crucial insight into the molecular profiles of entire PanINs and IPMNs. This will ideally allow researchers to characterize the precise transition points from LG to HG lesions, and similarly from HG lesions to PDAC. However, foundational to these studies is an important question: given the different clinical implications of LG versus HG lesions, what is the most beneficial time point along precursor progression at which to intervene (surgically or otherwise)? As with screening for most cancers and other diseases, the goal must be to maximize clinical benefit while minimizing harm from unnecessary intervention. Moving forward, research will focus on more precisely defining the molecular and cellular landscapes of PanINs and IPMNs as they progress to HG dysplasia and ultimately PDAC, with the goal of identifying more precise markers for pancreatic precancer interception.

## Version 1 07/15/2025

Electronic publication
